# Cardiac abnormalities in patients with septic shock detected by speckle tracking echocardiography

**DOI:** 10.1186/cc14215

**Published:** 2015-03-16

**Authors:** PY Ng, WC Sin, AK Ng, WM Chan

**Affiliations:** 1 Queen Mary Hospital, Hong Kong; 2 Grantham Hospital, Hong Kong

## Introduction

Sepsis-induced myocardial dysfunction is a well-recognized condition and confers worse outcomes in septic patients. However, the diagnostic criteria remain poorly described. Echocardiographic assessment by conventional parameters such as left ventricular ejection fraction (LVEF) is often affected by ongoing changes in preload and afterload conditions. Novel echocardiographic technologies such as speckle tracking imaging have evolved for direct assessment of the myocardial function. In this study, we investigate the measurement of myocardial strain by speckle tracking imaging for the diagnosis of sepsis-induced myocardial dysfunction.

## Methods

This is a prospective, case-control study at a university-affiliated tertiary care adult medical ICU. Consecutive patients admitted with a diagnosis of septic shock meeting the international consensus criteria were included. Patients with other causes of myocardial dysfunction were excluded. They are compared with age-matched, gender-matched, and cardiovascular risk factor-matched controls, who were admitted to hospital for sepsis but did not develop septic shock. Conventional echocardiographic parameters, as well as speckle tracking imaging of myocardial function, were obtained within 24 hours of diagnosis. Offline analyses of endocardial tracings were performed by two independent operators.

## Results

From January 2014 to December 2014, 32 patients with septic shock (study group) and 20 patients with sepsis but no septic shock (control group) were recruited. The baseline characteristics were similar. Conventional echocardiographic measurements, including LVEF (59.53% vs. 60.67% in the control group, *P *= 0.450) and fractional shortening (31.98% vs. 32.98%, *P *= 0.323), did not differ between the two groups. The study group had a greater degree of myocardial dysfunction measured by left ventricular global longitudinal strain (-14.6 vs. -17.6, *P *= 0.005, with a less negative value implying worse myocardial contractility). The hemodynamic profiles (cardiac index 3.49 l/minute/m^2^ vs. 3.41 l/minute/m^2^ respectively, *P *= 0.764) were not statistically different.

## Conclusion

This is a first study in the adult population to show that the use of speckle tracking imaging can diagnose significant sepsis-induced myocardial dysfunction, which was not otherwise detectable by conventional echocardiography.

